# Alcohol use as a predictor of child-to-parent violence in adolescents from southern Mexico

**DOI:** 10.1590/1980-220X-REEUSP-2024-0016en

**Published:** 2024-08-02

**Authors:** Pedro Moisés Noh-Moo, Lubia del Carmen Castillo-Arcos, Juan Yovani Telumbre-Terrero, Lucely Maas-Góngora, Sylvia Claudine Ramírez-Sánchez, Roberto Joel Tirado-Reyes

**Affiliations:** 1 Universidad Autónoma del Carmen, Facultad de Ciencias de la Salud, Ciudad del Carmen, Campeche, México.; 2 Instituto Mexicano del Seguro Social, Ciudad de México, Mexico.; 3 Universidad Autónoma de Sinaloa, Facultad de Enfermería Culiacan, Culiacán Rosales, Sinaloa, México.

**Keywords:** Alcohol, Domestic Violence, Adolescent, Álcool, Violência Doméstica, Adolescente

## Abstract

**Objective:**

To analyze the relationship and effect of alcohol use on Child-to-Parent Violence (CPV).

**Method:**

Cross-sectional, observational study with a quantitative approach, carried out through online data collection using the Conflict Tactics Scale (CTS2) and the Alcohol Use Disorders Identification Test (AUDIT) in 318 high school adolescents from southern Mexico.

**Results:**

Moderate and significant relationships were found between alcohol use and verbal (r_s_ = 0.408, p = 0.001) and economic violence against the mother (r_s_ = 0.445, p = 0.001). A similar situation is presented in physical (r_s_ = 0.473, p = 0.001), verbal (r_s_ = 0.236, p = 0.001) and economic (r_s_ = 0.477, p = 0.001) violence directed to the father.

**Conclusion:**

The relation among the variables was supported by Multiple Linear Regression models, with alcohol consumption in adolescents being a predictor of violence against mothers and fathers.

## RESUMO

**Objetivo:**

Analisar a relação e o efeito do uso de álcool na Violência Filioparental (VFP).

**Método:**

Estudo observacional transversal com abordagem quantitativa, realizado por meio de coleta de dados online usando a Escala Tática de Conflito (CTS2) e o Teste de Identificação de Transtornos por Uso de Álcool (AUDIT) em 318 adolescentes do ensino médio do sul do México.

**Resultados:**

Foram encontradas relações moderadas e significativas entre uso de álcool e violência verbal (r_s_ = 0,408, p = 0,001) e econômica cometida em relação à mãe (r_s_ = 0,445, p = 0,001). Uma situação semelhante é apresentada na violência física (r_s_ = 0,473, p = 0,001), verbal (r_s_ = 0,236, p = 0,001) e econômica (r_s_ = 0,477, p = 0,001) direcionada ao pai.

**Conclusão:**

A relação entre as variáveis foi apoiada por modelos de Regressão Linear Múltipla, sendo o consumo de álcool em adolescentes um preditor de violência contra mães e pais.

## INTRODUCTION

Alcohol is the most consumed legal drug in the world. Its abuse is the causal agent of approximately 200 pathologies, among which are non-communicable diseases, such as liver cirrhosis, some types of cancers, and cardiovascular system alterations. Moreover, it is associated with the development of mental and behavioral disorders, and is related to trauma of intentional or unintentional origin, particularly those due to traffic accidents, violence, and suicide. These diseases account for 5.1% of the global burden of disease, causing more than three million deaths per year (one every ten seconds)^(1)^.

One of the population groups with high prevalence of consumption are adolescents. Statistical analyses worldwide indicate that 27% of adolescents consume alcoholic beverages, with the region of the Americas having high proportions of consumption (38%)^(2)^. In the Mexican context, in 2022, the main national surveys reported that 20.6% of adolescents between 10 and 19 years old consumed alcohol in the past 12 months, of which 13.9% consume it excessively, with very small differences between men (22%) and women (19.2%)^(3)^. Studies carried out with students in the south and north areas of Mexico show prevalence of consumption at some point in their lives of 67.8% to 72.9%, with an average intake of 3 alcoholic beverages per consumption occasion^(4,5)^.

The previously cited statistics reveal that adolescents are a population segment who have the opportunity of trying and sustaining alcohol consumption, which is concerning, due to the stage of adolescence, where there is metabolic, neurocognitive and sensory-perceptive immaturity^(6)^. It has been observed that the use and abuse of alcohol allow for violent behavior that can have repercussions on the consumer’s family nucleus. Within this circle of family violence, it has been found that the two main axes are sex and age, with women and children being the main victimized population segments^(6,7)^. However, this role has been reversed, with the son or daughter being the one having a violent behavior towards their parents^(7)^.

In child-to-parent violence (CPV), it is the child who acts with the intention of achieving power, control, and dominance over his or her father or mother (or those who take their place) through physical (kicking, punching, and pushing), verbal (intimidation or threats, insults, screams), and economic (stealing of money, use of parents’ credit card, generating debts)^(7,8)^ attacks. Recently, it has been documented that attacks can be repeated and constantly established over time; that violence against the father or the mother can vary considerably according to the adolescents’ sex and age, with adolescence being a critical period, when most violent acts take place^(8)^.

Regarding substance use and CPV, studies carried out in the community or school population report that drug use is positively related with violence. In Canada, it was found that drug use increases the risk of verbal aggression towards the father and mother^(9,10)^. In Spain, the problematic drug abuse is positively related to psychological or verbal and physical violence^(11)^. In Colombia, it was found that adolescents exposed to drug use are at greater risk of stealing from their mothers^(12)^.

Moreover, a study carried out in the United States with a sample of incarcerated male and female adolescents found that attacks against parents occurred under the influence of drugs^(13)^. Other authors postulate the opposite, because substance use may be part of a coexisting pattern of antisocial behavior, as well as a psychoemotional maladjustment rather than a particular causal factor in CPV^(14,15)^. In addition, violent acts can be a response to the intergenerational transmission of family violence^(14,15)^.

In the field of drug addiction and CPV, there are still aspects that have not been explored; for instance, much of the scientific research in the last two decades has been carried out in Canada, Spain, the United States of America, England, Australia and New Zealand, showing a marked relationship with drug consumption, without explaining whether it correlates with any specific type of substance^(14,15)^. In Mexico, Latin America and the Caribbean literature on the subject is scarce. It should be mentioned that adolescents are exposed to opportunities of experiencing and sustaining unhealthy behaviors such as alcohol use and CPV. These behaviors can limit adolescents’ and parents’ life prospects, leading to losses and suffering in the family nucleus. This is the reason why the study represents an opportunity for nursing professionals and other health-related personnel to generate scientific evidence on this little-explored phenomenon, which will allow, in the near future, the design of priority multidisciplinary strategies and programs focused on the prevention of alcohol use and the different types of CPV, so that families have a healthy and risk-free relationship. The purpose of the present study was to analyze the relationship and effect of alcohol use on CPV.

## METHOD

### Design of Study

Observational study with a quantitative approach, carried out from August 2022− to December 2023. The survey was conducted online, and the criteria of the checklist for reporting results of Internet E-Surveys (CHERRIES) protocols were applied.

### Population and Sample

The population consisted of adolescents enrolled in high school. The sample was selected through a convenience procedure with open invitation through social media platforms (Facebook, Twitter and WhatsApp) that included a sample size (n = 318 adolescents) sufficient for the analysis, considering an alpha error of 0.05 and a power greater than 80%.

### Selection Criteria

Male and female adolescents from high school of a public institution in southern Mexico, ranging from 14 to 19 years, who answered the online survey through an open invitation, were included, totaling 318 participants, with an average age of 16.90 years (SD = 1.276).

### Data Collection

Data collection was carried out from January 2023 to March 2023. Recruitment of participants was carried out with an open schedule. This was disclosed on the main social media platforms of groups of students from all high school years where the research was developed. The instruments were programmed through the SurveyMonkey platform, the access link to the instruments was made available in the information systems and social media platforms of the public institution where the research was carried out. Sampling was suspended as soon as the sample had been established.

A total of 340 adolescents accessed the link. The digital survey programmed on the platform SurveyMonkey consisted of self-administered instruments, including a sociodemographic questionnaire prepared for this study, as well as the Conflict Tactics Scale (CTS2) – children to parents version by Strauss and Douglas^(16)^, adapted to Spanish by Gámez-Guadix et al.^(17)^, while the second questionnaire was the Alcohol Use Disorders Identification Test (AUDIT)^(18)^.

Information related to sex, age, occupation, school grade were taken from the personal data sheet. Participants were asked about the prevalence of use of alcoholic beverages at some point in life, throughout the past year, in the last 30 days, and during the previous seven days; they were also asked about the age of onset and the amount of alcoholic beverage intake.

The Conflict Tactics Scale (CTS2) allowed us to examine CPV through 20 items, divided into two subscales. The first is directed to the father, and the second to the mother. Both subscales evaluate the violence committed by the adolescent through situations experienced with parents. They respond independently, 10 items for father and mother. An example of a sentence described in the questionnaire is “I scream or have screamed at my parents.” Each item contains five Likert-like response options, ranging from 0 (never) to 4 (many times). The father and mother subscale is structured by three factors. The first factor is made up of items 3, 4, 5 and 6, addressing situations of physical violence. The second is characterized by verbal aggression assessment, and consists of items 1, 2 and 10. The third factor is structured by items 7, 8 and 9, encompassing stealing from parents (economic violence). The scale has been applied in a Latin American population^(12)^ and recently applied in adolescents from northern Mexico^(19)^, showing an internal consistency of α = 0.81 for the violence subscale directed towards the mother, while the subscale of violence against the father showed a reliability of α = 0.80. In this study, the global scale reported a Cronbach’s alpha of 0.75, the subscale of the father and mother showed reliability of α = 0.72 and α = 0.70, respectively.

To detect excessive consumption of alcoholic beverages during the past year, the Spanish version of AUDIT was used. This test consists of 10 items classified into three domains: risky alcohol consumption, dependence due to alcohol use, and harmful alcohol consumption. AUDIT scores range from 0 to 40 points. Therefore, for this study, the cut-off point is 8. In case the adolescent gets this score, he/she is considered as having harmful alcohol consumption. A score equal to or lower than 7 was considered risky alcohol consumption. The instrument has been applied to Mexican adolescents attending school in the south and north of Mexico, showing a reliability of α = 0.80 and α = 0.78^(4,5)^. In this study, the alcohol use detection test had a Cronbach’s Alpha of 0.85.

### Data Analysis and Treatment

Data obtained were analyzed using the Statical Package for Social Sciences (SPSS) version 24.0 for Windows. Descriptive analyses were carried out through frequencies and percentages, measures of central tendency, such as means and medians, were used, as well as variability, among which standard deviation stands out. The Chi Square test was used to compare alcohol use by sex. *Mann-Whitney U* was used to compare CPV scores by sex of the adolescent. Spearman’s correlation analysis was used to know the relationship between variables. Finally, a Multiple Linear Regression analysis with CPV as a dependent variable (physical, verbal and economic) was performed, with the AUDIT score as a predictor, meeting the assumptions of linearity through ANOVA (*p* < .05), the independence of the explanatory variables through the Durbin-Watson test (with scores ranging between 1.9 and 2.1), the homogeneity of the variances with the test of *Levene* (*p* < .05), and the degree of collinearity through the Variance Inflation Factor and tolerance that were between 1 and 0.1. As the variables did not present a normal distribution, the resampling technique bootstrap with 10,000 subsamples to calculate the 95% confidence intervals of the model^(20)^ was used.

### Ethical Considerations

The research was implemented after approval from the Research Ethics Committee of the Health Sciences School of the Universidad Autónoma del Carmen with registration number E2023F007, 2022, and from the educational institution where the study was carried out. The consents of the minor participants and the consent of their parents or guardians were obtained. Adolescents of legal age gave their own consent.

## RESULTS

Of the 318 participants, the vast majority were men (ƒ = 191; 60.1%), while only 39.9% (*F* = 127) were women. Three quarters of the sample were only dedicated to studying (73.9%, *F* = 235), of which 39% (ƒ = 124) were in the fourth semester, followed by those from the sixth semester (30.8%, ƒ = 98), and second semester (30.2%, ƒ = 96). Table 1 shows that 34.6% of the participants had consumed alcoholic beverages at some point in their lives, although a smaller number consumed it during the past week (3.1%). The adolescents started drinking alcohol around the age of 16.09 (SD = 1.57), with women consuming 4.85 (SD = 2.303) alcoholic drinks per consumption occasion and men around 5.10 alcoholic drinks (SD = 2.385). Statistically significant differences were identified in the prevalence of alcohol use at some point in life, in the last year and in the last seven days, with women having the highest proportion of consumption (*p < .05*).

**Table 1:** Alcohol use by adolescent’s sex – Carmen, Campeche, Mexico, 2023.

Prevalence	n = 318		Sex				*χ* ^2^	*p*
	Total sample		Female		Male			
	*f*	%	*f*	%	*f*	%		
Sometime in life								
Consumption	110	34.6	59	53.6	51	46.4	13.157	.001
No consumption	208	65.4	51	32.7	140	67.3		
In the past year								
Consumption	70	22	38	54.3	32	45.7	7.704	.006
No consumption	248	78	89	35.9	159	64.1		
In the last 30 days								
Consumption	43	13.5	21	48.8	22	51.2	1.642	.200
No consumption	275	86.5	106	38.5	169	39.5		
In the past week								
Consumption	10	3.1	9	90	1	10	10.788	.001
No consumption	308	96.9	118	38.3	190	61.7		

Note: ƒ = Frequencies, % = Percentages, χ^2^ = Chi square, *p* = Statistical significance.

Source: Survey data.

The adolescents obtained 5.457 (SD = 4.37) points through the AUDIT test. Table 2 indicates that 16.4% of adolescents who consumed alcoholic beverages during the past year had risky consumption, while 5.7% had harmful consumption. Women presented a high proportion of risky alcohol consumption. However, harmful alcohol consumption predominates in men (X^2^ = 4.286, *p* = .038).

**Table 2:** Alcohol use according to the AUDIT – Carmen, Campeche, Mexico, 2023.

Pattern	Global		Women		Men	
	*f*	%	*f*	%	*f*	%
No consumption in the past year	248	78	89	35.9	159	64.1
Risky alcohol consumption	52	16.4	32	61.5	20	38.5
Harmful consumption	18	5.6	6	33.3	12	66.7

Notes: ƒ = Frequencies, % = Percentages.

Source: Survey data.

The participants had 3,349 points (SD = 3.993) in the subscale of violence directed towards the father and 2 points (SD = 2.703) in the subscale of violence directed towards mothers. The violence that is most frequently exerted on both mothers and fathers is verbal violence (*M* = 1.50 and *M* = 1.51), followed by the economic (*M* = 0.95 and *M* = 0.92) and the physical ones (*M* = 0.39 and *M* = 0.90) respectively. Table 3 shows that male adolescents, compared to females, use greater verbal (*M* = 1.75, *p* = .001) and economic (*M* = 1.35, *p* = .001) violence towards mothers, while towards fathers they are directed with greater physical (*M* = 1.30, *p* = .001), verbal (*M* = 1.76, *p* = .001), and economic (*M* = 1.38, *p* = .001) violence. There were no statistical differences regarding physical CPV. In the results presented in Table 4, a positive and significant relationship can be seen between alcohol use and the CPV factors of both the father and the mother (*p* < .05), except for the factor of physical violence directed towards the mother (*p* > .05). The results that were significant indicate that in any of the situations, the greater the use of alcohol, the greater the violence towards both parents.

**Table 3:** CPV depending on the sex of the adolescent – Carmen, Campeche, Mexico, 2023.

Adolescent								
	Female			Male			*U*	*p*
	*M*	*Mdn*	*SD*	*M*	*Mdn*	*SD*		
Mother								
Physical	.409	.000	.937	.387	.000	.825	12071.5	.923
Verbal	1.13	1.00	1.22	1.75	2.00	1.35	8811.00	.001
Economic	.259	.000	.768	1.40	1.0	1.4	6453.00	.001
Father								
Physical	.401	.000	.986	1.24	1.00	1.30	7050.00	.001
Verbal	.818	.000	1.28	1.97	2.00	1.76	7418.00	.001
Economic	.236	.000	.840	1.38	1.00	1.62	6709.00	.001

Note: *M* = *Mean*, *Mdn* = Median, SD *=* Standard deviation, *U = Mann-Whitney U*, *p* = Statistical significance, *n* = 318.

Source: Survey data.

**Table 4:** Spearman’s Correlation matrix of alcohol consumption and CPV – Ciudad del Carmen, Campeche, Mexico, 2023.

Violence	Audit
Mother	
Physical	.231
Verbal	.408**
Economic	.445**
Father	
Physical	.473**
Verbal	.236**
Economic	.477**

Note:

** *p* <.001,

*p < .05.

Source: Survey data.

Multiple linear regression analyses were performed (maintaining the age of the participants as a confounding variable) based on the sex of the parents, with the CPV factors as the dependent variable and the AUDIT score as the independent variable (Table 5). In the first model related to mothers, the equation was not significant (*F* = 2.475, *p* = 0.092) and it can be seen that the predictors of physical violence were not reported to be significant (*p* > 0.05). However, in verbal violence model 2 was significant (*F* = 8,916, *p* = 0.001), explaining 21% of the variation in the dependent variable. The AUDIT score was shown to be a predictor of verbal violence (*b* = 0.142, *p* = 0.001). Regarding the prediction of economic violence, model 3 was significant (*F* = 6,280, *p* = 0.003), with only the AUDIT score being a predictive variable (*b* = 0.087, *p* = 0.014), with 15.8% explanation of the variance.

**Table 5:** Prediction estimators of CPV against parents – Carmen, Campeche, Mexico, 2023.

Violence against mothers (Model 1) Physical	*B*	*t*	*p*	Confidence interval at 95% of BCa	
				Lower Limit	Upper Limit
Age	–.250	–1.776	1.33	–.605	.029
AUDIT	0.62	1.815	.114	–.007	.151
(Model 2) Verbal					
Age	.066	.434	.680	–.262	.324
AUDIT	.142	3.872	.001	.044	.241
(Model 3) Economic					
Age	1.80	1.361	.212	–.081	.439
AUDIT	.087	2.707	.014	.029	.271
Violence against parents (Model 1) Physics					
Age	.417	2.60	.009	.152	.687
AUDIT	.113	.297	.011	.044	.208
(Model 2) Verbal					
Age	.397	2.167	.037	.061	.688
AUDIT	.152	3.435	.004	.056	.253
(Model 3) Economic					
Age	.437	3.055	.015	.171	.714
AUDIT	.135	3.891	.005	.065	.223

Note: *B* =Beta, *t* = *t* Value, *p* = Statistical significance, *BCa* = Bias-corrected and accelerated bootstrap based on 10000 samples.

Source: Survey data.

Regarding parents, the predictive estimators of the CPV factors were significant. Regarding the prediction of physical violence, the first model was reported to be significant (*F* = 11,008, *p* = 0.001), explaining 24.7% of the variance of the dependent variable. Age (*B* = .417, *p* = .009) and the AUDIT score (*b* = .113, *p* = .011) are significant predictors. In the case of model 2 on verbal violence, it showed to be significant (*F* = 11,561, *p* = 0.001), with an explanatory variance of 25.7% on the dependent variable, with age and AUDIT score being significant predictors. This same situation occurred in model 3 of economic violence (*F* = 17,432, *p* = 0.001), although with an explanatory variance of 34.2% on the dependent variable. In any of the situations, the results are related with greater age and involvement with alcohol use, leading to greater physical, verbal and economic violence against parents.

## DISCUSSION

The findings obtained in the present study show that the prevalence of alcohol consumption in high school adolescents tends to increase. In addition, the reported percentages of consumption by the female sex are above what was documented by studies in the Mexican context and by those carried out in a similar population. The increase in consumption among women could be attributed to changes in social norms, partly linked to advances in gender equality, where risky behaviors of the female sex are currently accepted and less stigmatized by society^(3–5,19)^. Furthermore, the significant increase in alcohol consumption in both sexes could be explained by the poor supervision of the sale and distribution of this substance, along with the low risk perception they have towards alcohol use^(3,19)^, something that has important implications at a preventive level, highlighting the need for work in early adolescence (particularly in high school adolescents), since the early onset of alcohol consumption can have serious repercussions on the adolescent’s health and life.

Regarding CPV by adolescents towards their parents, it was found that both sexes commit violence, but this varies depending on the sex of the adolescent. It was expected that women would direct more verbal and economic violence towards mothers and men would practise greater physical violence towards both parents^(21)^. In the present study, an important tendency was found towards verbal violence, followed by economic and physical violence by the male sex, with fathers being the main recipients of violent acts. The findings indicate that Mexican families are undergoing changes in parenting styles, from authoritarian families (where the father is the head of the home) to permissive-liberal families, who are less strict and have little affection (neglectful), with no clear rules established nor a consistent limit of authority^(22)^. In permissive and negligent families, it has been observed that children are overprotected and have little empathy; they are given what they ask but are not demanded for anything; therefore, when children’s demands are not satisfied, they assume antisocial behavior and, many times, violent verbal and physical behavior and even theft towards parents to obtain what is requested^(22,23)^.

The main objective of this study was to analyze the relationship and effect of alcohol use on CPV. The findings reproduce results from previous studies carried out in different national contexts, although it should be noted that the use of alcohol by Mexican adolescents was positively related to verbal and economic violence directed to mothers, while towards fathers it was positively related with the three CPV factors. Likewise, the use of alcohol through the regression models was found to explain CPV factors against mothers and fathers, demonstrating that the use of this substance increases violence towards parents. On the other hand, age was a complementary explanatory component only of the violence committed against fathers, indicating that the higher the age, the greater the aggression.

Based on the data obtained on the relationship between alcohol use and CPV, it can be said that adolescents who consume alcohol show greater violence towards both parents. Following this line, several investigations have emphasized the implication of substance use with CPV and their results show consistent positive relationships between substance use and CPV factors in the adolescent population, although there are few studies linked only to the specific use of alcohol^(24)^. In this regard, violence carried out by adolescents under the influence of a substance can be explained from an instrumental or reactive reason^(25)^. In the instrumental reason, when the adolescent is addicted to the substance, he or she compulsively searches for it, despite its harmful consequences^(26)^. Since parents are the financial source for the purchase of alcohol, and given the refusal by the father and the mother to meet their child’s demand, attacks can be triggered that will possibly increase as dependence and the combination of other drugs increase. Therefore, alcohol abuse is a risk factor for aggressive behavior towards father and mother^(25,26)^.

The reactive reason can be explained as a product of the verbal and behavioral disinhibition that the substance causes in the adolescent, increasing the risk of physical aggression escalation^(25,26)^. Therefore, the findings suggest that alcohol consumption is a trigger for the violence that occurs in many families, where there is fear of the son and daughter and of the possible physical injuries that they may cause, sometimes being silenced because they become patterns that are tolerated^(26)^.

Regarding the particularity of the types of violence, the regression models showed that alcohol use predicts verbal and economic violence on mothers. This shows that mothers are possibly violated because they are considered the weaker sex and due to the closeness they have with their children, which puts them in a position of risk^(27)^. Moreover, in Mexico there are changes in family models (decrease in nuclear families), around 10 million families are headed by women^(28)^, in such a way that since they are the home heads, they need to work to support the family and, as a consequence, they limit contact with their children, losing the maintenance of supervision and authority, which can lead the son to adopt unhealthy behaviors such as the consumption of alcoholic beverages. Since mothers are the source of money to buy alcohol, when they do not provide it, verbal attacks and even theft can arise^(27,28)^.

Thus, alcohol use and age were significant predictors of physical, verbal, and economic violence against parents. Regarding violence, results are partially consistent with previous studies of the Mexican context where prediction of verbal violence towards parents^(19)^ was found. In Germany, substance use predicted male adolescents’ physical violence against their parents^(29)^. The results show that both alcohol consumption and violence have a significant connection within the family nucleus and are often patterns that are repeated between parents and male children. Currently, the permissive and negligent parenting style on the part of the father can be affecting adolescents’ behavior, given that the roles are increasingly more symmetrical, with the parental figure losing authority^(19,29)^.

Regarding age, the findings contribute to previous studies, which point out that the peak of violence is between 14 and 16 years (middle adolescence), gradually decreasing as age progresses. However, the present study addressed adolescents between 14 and 19 years of age, finding a positive relation between age and CPV, indicating that the older the age, the more severe the violence. These findings show that CPV not only appears during adolescence but can also be present in other stages of life, such as the youth^(19,29,30)^.

The study has some limitations: sample selection through non-probabilistic sampling and the sample size. Both limitations prevent the generalization of the results obtained and could explain the low variance of the dependent variable. Other limitations observed are the non-normal distribution of the variables, which could limit the predictive estimation of the model on the dependent variable, and the nature of the design, which is cross-sectional, thus hindering establishment of causality. It should be mentioned that the origin of the adolescents was not identified, which limits the significant representation of each region that makes up southern Mexico. In future research, the expansion of the sample size and application of probabilistic sampling to establish significant representation in the sample are suggested. The observation of the relation between alcohol consumption within underage and adult adolescents (or by age group) and CPV is also recommended, as well as the integration of other variables that may influence the appearance of the CPV. Based on this, a model with strong methodological rigor can be constructed.

## CONCLUSION

CPV is still a hidden and stigmatized issue among Mexican families. The results confirm the relationship and effect of alcohol consumption on CPV, demonstrating that the implication of alcohol use by adolescents can modify family dynamics and generate situations of conflict such as violent behavior within the family nucleus (from the son and daughter towards the parents).

Accordingly, the nursing professional must intervene through the design and implementation of nursing interventions aimed at preventing or reducing the use of alcohol and different types of CPV. Furthermore, the nursing professional, from his field of action at the first level of care, can intervene by identifying risk factors for the beginning of alcohol consumption, as well as detect patterns of violent behavior in the son and daughter towards the father or mother and, based on this, promote protective parenting styles, encourage assertive or healthy communication in adolescents and parents to prevent risk behaviors among members of the family nucleus.
