# Supplementary material for: Alcohol use as a predictor of child-to-parent violence in adolescents from southern Mexico
Source: Rev Esc Enferm USP. 2024 Aug 2;58:e20240016. doi: 10.1590/1980-220X-REEUSP-2024-0016en (PMC11321823; doi:10.1590/1980-220X-REEUSP-2024-0016en)
Supplement: Translation

# Translation: Uso de alcohol como predictor de la violencia filio-parental en adolescentes del sur de México

**DOI:** 10.1590/1980-220X-REEUSP-2024-0016es

**Authors:** Pedro Moisés Noh-Moo, Lubia del Carmen Castillo-Arcos, Juan Yovani Telumbre-Terrero, Lucely Maas-Góngora, Sylvia Claudine Ramírez-Sánchez, Roberto Joel Tirado-Reyes

**Affiliations:** 1 Universidad Autónoma del Carmen, Facultad de Ciencias de la Salud, Ciudad del Carmen, Campeche, México.; 2 Instituto Mexicano del Seguro Social, Ciudad de México, Mexico.; 3 Universidad Autónoma de Sinaloa, Facultad de Enfermería Culiacán, Culiacán Rosales, Sinaloa, México.

**Keywords:** Alcohol, Violencia Doméstica, Adolescente

## Abstract

**Objetivo:**

Analizar la relación y efecto del uso de alcohol sobre la Violencia Filio-Parental (VFP).

**Método:**

Estudio observacional transversal con enfoque cuantitativo, realizado mediante recolección de datos en línea utilizando la Escala Táctica de Conflicto (CTS2) y la Prueba de Identificación de Desordenes por Uso de Alcohol (AUDIT) en 318 adolescentes de preparatoria del sur de México.

**Resultados:**

Se encontraron relaciones moderadas y significativas entre el uso de alcohol y la violencia verbal (r_s_ = .408, p = .001) y económica ejercida hacia la madre (r_s =_ .445, p = .001). Situación similar se presentó en la violencia física (r_s_ = .473, p = .001), verbal (r_s_ = .236, p = .001) y económica (r_s_ = .477, p = .001) dirigida al padre.

**Conclusión:**

La relación entre las variables fue respaldada por modelos de Regresión Lineal Múltiple, siendo el consumo de alcohol en los adolescentes predictor de la violencia ejercida a las madres como a los padres.

## INTRODUCCIÓN

El alcohol es la droga legal de mayor consumo en el mundo, su abuso es agente causal de aproximadamente 200 patologías, entre las que se encuentran padecimientos no transmisibles, como cirrosis hepática, algunos tipos de cánceres y alteraciones del sistema cardiovascular. Asimismo, está asociado con el desarrollo de trastornos mentales y comportamentales; también se relaciona con traumatismos de origen intencional o no intencional, en particular los debidos a accidentes de tránsito, violencia y suicidios. Dichos padecimientos ocupan el 5.1% de la carga mundial de morbilidad, ocasionando más de tres millones de muertes al año (uno cada diez segundos)^(1)^.

Uno de los grupos poblacionales con prevalencias altas de consumo son los adolescentes. Los análisis estadísticos a nivel mundial indican que una cuarta parte (27%) de los adolescentes consumen bebidas alcohólicas, siendo la región de las Américas la que presenta altas proporciones de consumo (38%)^(2)^. En el contexto mexicano, durante el 2022, las principales encuestas nacionales reportaron que el 20.6% de los adolescentes de 10 a 19 años consumieron alcohol en los últimos 12 meses, de los cuales el 13.9% lo consumen de manera excesiva, con diferencias muy pequeñas entre hombres (22%) y mujeres (19.2%)^(3)^, mientras que estudios realizados con estudiantes en la zona sur y norte de México muestran prevalencias de consumo alguna vez en la vida de 67.8% a 72.9%, con un promedio de ingesta de 3 bebidas alcohólicas por ocasión de consumo^(4,5)^.

Las estadísticas previamente citadas revelan que los adolescentes son un segmento poblacional con oportunidad de experimentar y sostener el consumo de alcohol, lo cual es preocupante debido a la etapa de la adolescencia, donde existe inmadurez metabólica, neurocognitiva y sensoperceptiva^(6)^. Se ha observado que el uso y abuso de alcohol posibilita conductas violentas que pueden repercutir en el núcleo familiar del consumidor. Dentro de este círculo de violencia familiar, se ha constatado que los dos principales ejes lo constituyen el sexo y la edad, destacando a las mujeres y los niños los principales segmentos poblacionales victimizados^(6,7)^. Sin embargo, hace algunos años este rol se ha invertido, siendo el hijo o la hija quien ejerce conductas violentas a sus progenitores^(7)^.

En la violencia filio-parental (VFP) es el/la hijo/a quien actúa con la intención de conseguir poder, control y dominio sobre su padre o madre (o quienes ocupen su lugar) a través de agresiones físicas (patadas, puñetazo y empujones), verbales (intimidaciones o amenazas, insultos, gritos) y económicas (robo de dinero, utilizar tarjeta de crédito de los padres para generar deudas)^(7,8)^. Recientemente se documentó que las agresiones se pueden repetir y establecerse de manera constante a lo largo del tiempo, además que la violencia ejercida contra el padre o la madre pueden variar considerablemente por el sexo y la edad de los adolescentes, siendo la adolescencia un período crucial donde se presentan la mayor parte de los actos violentos^(8)^.

Respecto al uso de sustancias y la VFP, investigaciones realizadas en población comunitaria o escolarizada reportan que el uso de drogas se relaciona de manera positiva. En Canadá se encontró que el consumo de drogas aumenta el riesgo de agresión verbal hacia el padre y la madre^(9,10)^. En España se identificó que el abuso problemático de drogas se relaciona de manera positiva con la violencia de tipo psicológica o verbal y física^(11)^. En Colombia se identificó que los adolescentes expuestos al uso de drogas presentan un riesgo más elevado de robar a la madre^(12)^.

Un estudio efectuado en Estados Unidos con muestra de adolescentes hombres y mujeres encarcelados identificó que las agresiones a los progenitores ocurrieron bajo el efecto de las drogas^(13)^. Otros autores postulan lo contrario, debido que el uso de sustancias puede ser parte de un patrón coexistente de comportamiento antisocial, así como de un desajuste psicoemocional en lugar de un factor causal particular en la VFP^(14,15)^. Además, los actos violentos pueden ser una respuesta a la transmisión intergeneracional de la violencia familiar^(14,15)^.

En el campo de las drogodependencias y la VFP, aún existen aspectos pocos explorados, por ejemplo, gran parte de las investigaciones científicas en las dos últimas décadas se han desarrollado en Canadá, España, Estados Unidos de América, Inglaterra, Australia y Nueva Zelanda, mostrando una marcada relación con el consumo de drogas, sin explicar si correlaciona con algún tipo de sustancia en específico^(14,15)^. En México, Latinoamérica y el Caribe existe escasa literatura sobre el tema. Es importante mencionar que los adolescentes están expuestos a oportunidades para experimentar y sostener conductas no saludables como el uso de alcohol y la VFP. Dichas conductas pueden limitar la proyección de vida de los adolescentes y padres de familia, ocasionando pérdidas y sufrimiento en el núcleo familiar, razón por la cual el estudio representa oportunidad para que el profesional de enfermería y personal sanitario afines generen evidencia científica sobre este fenómeno poco explorado, que permita en un futuro cercano el diseño de estrategias y programas prioritarios multidisciplinares enfocados a la prevención del uso de alcohol y los diferentes tipos de VFP, de manera que las familias tengan una relación saludable y libre de riesgos. El propósito del presente estudio fue analizar la relación y efecto del uso de alcohol sobre la VFP.

## MÉTODO

### Diseño de Estudio

Estudio observacional con enfoque cuantitativo, llevado a cabo de agosto 2022 hasta diciembre 2023. La encuesta se realizó en línea, y se aplicaron los criterios de la lista de verificación para el reporte de resultados de protocolos de Internet E-Surveys (CHERRIES).

### Población y Muestra

La población estuvo conformada por adolescentes escolarizados de preparatoria. La muestra se seleccionó mediante un procedimiento a conveniencia con invitación abierta a través de redes sociales (Facebook, Twitter y WhatsApp) que integró un tamaño de muestra (n = 318 adolescentes) suficiente para el análisis, considerando un error de alfa de 0.05 y una potencia superior al 80%.

### Criterios de Selección

Se integraron adolescentes hombres y mujeres de una preparatoria pública del sur de México, con rango de edad de 14 a 19 años, que contestaron la encuesta en línea mediante invitación abierta, obteniendo 318 participantes, con promedio de edad de 16.90 años (DE = 1.276).

### Colecta de Datos

La recolección de datos se efectuó durante el periodo de enero 2023 hasta marzo 2023. El reclutamiento de los participantes se llevó a cabo con un horario abierto. Se realizó divulgación en las principales redes sociales de grupos de estudiantes de todos los semestres de la preparatoria donde se desarrolló la investigación. Los instrumentos se programaron a través de la plataforma SurveyMonkey, el enlace de acceso a los instrumentos se puso a disposición en los sistemas de información y redes sociales de la institución pública donde se efectuó la investigación, el muestreo se detuvo hasta que se integró la muestra establecida.

En total accedieron al enlace 340 adolescentes, la encuesta digital programada en la plataforma SurveyMonkey estuvo constituida por instrumentos autoadministrados, y consistió en un cuestionario sociodemográfico elaborado para este estudio, así como de la Escala Táctica de Conflicto (CTS2) – versión hijos a padres de Strauss y Douglas^(16)^ adaptada al español por Gámez-Guadix et al.^(17)^, mientras que el segundo cuestionario fue la Prueba de Identificación de Desordenes por Uso de Alcohol (AUDIT)^(18)^.

En el formulario de datos personales se recopiló información relacionada al sexo, edad, ocupación, semestre escolar, además de indagar sobre la prevalencia del uso de bebidas alcohólicas en algún momento de la vida, durante el último año, en los últimos 30 días y durante los últimos siete días; también se indagó sobre la edad de inicio y cantidad de ingesta de bebidas alcohólicas por ocasión de consumo.

La Escala Táctica de Conflicto (CTS2) permitió examinar la VFP a través de 20 ítems, divididos en dos subescalas. La primera va dirigida al padre, y la segunda va dirigida a la madre. Ambas subescalas evalúan la violencia cometida por el adolescente a través de situaciones vividas con los progenitores. Se responden de forma independiente, 10 ítems para el padre y la madre. Un ejemplo de una frase descrita en el cuestionario es “grito o he gritado a mis padres”. Cada ítem contiene cinco opciones de respuesta tipo Likert, que van desde 0 (nunca) hasta 4 (muchas veces). La subescala del padre y la madre está estructurada por tres factores. El primer factor está constituido por los ítems 3, 4, 5 y 6, aborda situaciones sobre violencia física. El segundo se caracteriza por evaluar agresiones de tipo verbal, está integrada por los ítems 1, 2 y 10. El tercer factor está estructurado por los ítems 7, 8 y 9, engloba situaciones de robo a los progenitores (violencia económica). La escala ha sido aplicada en población Latinoamericana^(12)^ y recientemente aplicada en adolescentes del norte de México^(19)^ mostrando una consistencia interna de α = .81 para la subescala de violencia dirigida a la madre, mientras que en la subescala de violencia al padre reportó una fiabilidad de α = .80. En este estudio, la escala global reportó un Alpha de Cronbach de .75, la subescala del padre y la madre arrojaron una confiabilidad de α = .72 y α = .70, respectivamente.

Para detectar el consumo excesivo de bebidas alcohólicas durante el último año se empleó el AUDIT en versión español. Esta prueba está integrada por 10 ítems clasificados en tres dominios: consumo de riesgo de alcohol, dependencia por uso de alcohol y consumo perjudicial de alcohol. Los puntajes del AUDIT van de 0 a 40 puntos, de tal manera que para este estudio el punto de corte es 8. Si el adolescente obtiene dicha puntuación, se identificó con un consumo perjudicial de alcohol, en caso que obtenga una puntuación igual o menor a 7, se consideró consumo riesgoso de alcohol. El instrumento ha sido aplicado en adolescentes mexicanos escolarizados del sur y norte de México mostrando una fiabilidad de α = .80 y α = .78^(4,5)^. En este estudio la prueba de detección de uso de alcohol tuvo un Alpha de Cronbach de .85.

### Análisis y Tratamiento de Los Datos

Los datos obtenidos fueron analizados por medio del Statical Package for Social Sciences (SPSS) versión 24.0 para Windows. Se ejecutaron análisis descriptivos a través de frecuencias, porcentajes; se utilizaron medidas de tendencia central como medias y medianas, así como de variabilidad, entre la que destaca desviación estándar. Se utilizó prueba de Chi Cuadrada para comparar por sexo el uso de alcohol, se aplicó el estadístico de *U de Mann-Whitney* para comparar los puntajes de VFP por sexo del adolescente, se recurrió al análisis de Correlación de *Spearman* para conocer la relación entre variables. Finalmente se realizó análisis de Regresión Lineal Múltiple sobre la VFP como variable dependiente (física, verbal y económica), teniendo como predictor la puntuación AUDIT, cumpliendo los supuestos de linealidad a través de ANOVA (*p* < .05), la independencia de las variables explicativas por medio la prueba Durbin-Watson (con puntuaciones que oscilaron entre 1.9 y 2.1), la homogeneidad de las varianzas con el test de *Levene* (*p* < .05) y el grado de colinealidad a través de la prueba de Tolerancia y Factor de Inflación de la Varianza (VIF) que se ubicaron entre 1 y 0.1, debido a que las variables no presentaron distribución normal se recurrió a la técnica de remuestreo *bootstrap* con 10000 submuestras para calcular los intervalos de confianza del modelo al 95%^(20)^.

### Consideraciones Éticas

La investigación se implementó con la obtención de la aprobación del comité de Ética en Investigación de la Facultad de Ciencias de la Salud de la Universidad Autónoma del Carmen con número de registro E2023F007 de 2022, así como de la institución educativa donde se efectuó el estudio, se contó con el asentimiento para el participante menor de edad y con el consentimiento de los padres o tutores de estos mismos adolescentes; para el adolescente mayor de edad, se obtuvo su consentimiento.

## RESULTADOS

De los 318 participantes, la gran mayoría fueron hombres (ƒ = 191; 60.1%), mientras que solo el 39.9% (*f* = 127) mujeres. Tres cuartas partes de la muestra solo se dedicaba a estudiar (73.9%, *f* = 235), de los cuales el 39% (ƒ = 124) se encontraban en el cuarto semestre, seguido del sexto semestre (30.8%, ƒ = 98) y segundo semestre (30.2%, ƒ = 96). La Tabla 1 muestra que el 34.6% de los participantes presentaron ingesta de bebidas alcohólicas en algún momento de su vida, aunque en menor cantidad lo consumieron durante la última semana (3.1%). Los adolescentes iniciaron a consumir alcohol alrededor de los 16.09 años (*DE* = 1.57), consumiendo las mujeres 4.85 (*DE* = 2.303) bebidas alcohólicas por ocasión de consumo y los hombres alrededor de 5.10 bebidas alcohólicas (*DE* = 2.385). Se identificaron diferencias estadísticamente significativas en la prevalencia del uso de alcohol en algún momento de la vida, en el último año y en los últimos siete días, siendo las mujeres con mayor proporción de consumo (*p < .05*).

**Tabla 1:** Uso de alcohol por sexo del adolescente – Carmen, Campeche, México, 2023.

Prevalencia	n = 318		Sexo				*χ* ^2^	*p*
	Muestra total		Femenino		Masculino			
	*f*	%	*f*	%	*f*	%		
Alguna vez en la vida								
Sí consumió	110	34.6	59	53.6	51	46.4	13.157	.001
No consumió	208	65.4	51	32.7	140	67.3		
En el último año								
Sí consumió	70	22	38	54.3	32	45.7	7.704	.006
No consumió	248	78	89	35.9	159	64.1		
En los últimos 30 días								
Sí consumió	43	13.5	21	48.8	22	51.2	1.642	.200
No consumió	275	86.5	106	38.5	169	39.5		
En la última semana								
Sí consumió	10	3.1	9	90	1	10	10.788	.001
No consumió	308	96.9	118	38.3	190	61.7		

Note: ƒ = Frecuencias, % = Porcentajes, χ^2^ = Chi cuadrada, *p* = Significancia estadística.

Fuente: Datos de la encuesta.

Los adolescentes obtuvieron 5.457 (DE = 4.37) puntos a través de la prueba AUDIT. La Tabla 2 indica que el 16.4% de los adolescentes que tuvieron ingesta de bebidas alcohólicas durante el último año presentaron un consumo riesgoso, mientras que el 5.7% se ubicó en un consumo perjudicial. Las mujeres presentan alta proporción de consumo riesgoso de alcohol, sin embargo, en los hombres predomina el consumo perjudicial de alcohol (*X* ^2^ = 4.286, *p* = .038).

**Tabla 2:** Uso de alcohol de acuerdo al AUDIT – Carmen, Campeche, México, 2023.

Patrón	Global		Mujeres		Hombres	
	*f*	%	*f*	%	*f*	%
Sin consumo en el último año	248	78	89	35.9	159	64.1
Consumo riesgoso de alcohol	52	16.4	32	61.5	20	38.5
Consumo perjudicial	18	5.6	6	33.3	12	66.7

Notes: ƒ = Frecuencias, % = Porcentajes.

Fuente: Datos de la encuesta.

Los participantes tuvieron 3.349 puntos (*DE* = 3.993) en la subescala de violencia dirigida a los padres y 2 puntos (*DE* = 2.703) en la subescala de violencia dirigida a las madres. La violencia que más se ejerce tanto en las madres como en los padres es la violencia de tipo verbal (*M* = 1.50 y *M* = 1.51), seguida de la económica (*M* = 0.95 y *M* = 0.92) y física (*M* = 0.39 y *M* = 0.90) respectivamente. En la Tabla 3 se muestra que los adolescentes del sexo masculino en comparación con las mujeres se dirigen con más violencia verbal (*M* = 1.75, *p* = .001) y económica (*M* = 1.35, *p* = .001) a las madres, mientras que al padre se dirigen con mayor violencia física (*M* = 1.30, *p* = .001), verbal (*M* = 1.76, *p* = .001) y económica (*M* = 1.38, *p* = .001). No hubo diferencias estadísticamente respecto a la VFP física.

**Tabla 3:** VFP en función del sexo del adolescente – Carmen, Campeche, México, 2023.

Adolescente								
	Mujer			Hombre			*U*	*p*
	*M*	*Mdn*	*DE*	*M*	*Mdn*	*DE*		
Madre								
Física	.409	.000	.937	.387	.000	.825	12071.5	.923
Verbal	1.13	1.00	1.22	1.75	2.00	1.35	8811.00	.001
Económica	.259	.000	.768	1.40	1.0	1.4	6453.00	.001
Padre								
Física	.401	.000	.986	1.24	1.00	1.30	7050.00	.001
Verbal	.818	.000	1.28	1.97	2.00	1.76	7418.00	.001
Económica	.236	.000	.840	1.38	1.00	1.62	6709.00	.001

Note: *M* = Media, *Mdn* = Mediana, *DE* = Desviación estándar, *U = U de Mann-Whitney*, *p* = Significancia estadística, *n* = 318.

Fuente: Datos de la encuesta.

En los resultados presentados en la Tabla 4 se logra apreciar relación positiva y significativa entre el uso de alcohol y los factores de VFP tanto del padre como de la madre (*p* < .05), a excepción del factor de violencia física dirigida a la madre (*p* > .05). Los resultados que fueron significativos indican que en cualquiera de las situaciones cuanto más uso de alcohol más elevada es la violencia con ambos progenitores.

**Tabla 4:** Matriz de correlación de Spearman del consumo de alcohol y la VFP – Ciudad del Carmen, Campeche, México, 2023.

Violencia	AUDIT
Madre	
Física	.231
Verbal	.408**
Económica	.445**
Father	
Física	.473**
Verbal	.236**
Económica	.477**

Note:

** *p* <.001,

*p < .05.

Fuente: Datos de la encuesta.

Se ejecutaron análisis de regresión lineal múltiple (manteniendo como variable confusora la edad de los participantes) en función del sexo de los padres, teniendo como variable dependiente los factores de la VFP y como variable independiente la puntuación AUDIT (Tabla 5). En el primer modelo sobre las madres, la ecuación no se mostró significativa (*F* = 2.475, *p* = .092) y se logra apreciar que los predictores de violencia física no se reportaron significativos (*p* > .05). Sin embargo, en la violencia verbal, el modelo 2 fue significativo (*F* = 8.916, *p* = .001), explicando el 21% de la variación de la variable dependiente. La puntuación AUDIT se mostró predictora de la violencia verbal (*B* = .142, *p* = .001). En cuanto a la predicción de la violencia económica, el modelo 3 resultó significativo (*F* = 6.280, *p* = .003), siendo únicamente la puntuación AUDIT variable predictiva (*B* = .087, *p* = .014), con 15.8% de explicación de la varianza.

**Tabla 5:** Estimadores de predicción de la VFP contra los progenitores – Carmen, Campeche, México, 2023.

Violencia contra las madres (Modelo 1) Física	*B*	*t*	*p*	Intervalo de Confianza al 95% de BCa	
				Límite Inferior	Límite Superior
Edad	–.250	–1.776	1.33	–.605	.029
AUDIT	0.62	1.815	.114	–.007	.151
(Modelo 2) Verbal					
Edad	.066	.434	.680	–.262	.324
AUDIT	.142	3.872	.001	.044	.241
(Modelo 3) Económica					
Edad	1.80	1.361	.212	–.081	.439
AUDIT	.087	2.707	.014	.029	.271
Violencia contra los padres (Modelo 1) Física					
Edad	.417	2.60	.009	.152	.687
AUDIT	.113	.297	.011	.044	.208
(Modelo 2) Verbal					
Edad	.397	2.167	.037	.061	.688
AUDIT	.152	3.435	.004	.056	.253
(Modelo 3) Económica					
Edad	.437	3.055	.015	.171	.714
AUDIT	.135	3.891	.005	.065	.223

Note: *B* =Beta, *t* = *t* Valor, *p* = Significancia estadística, *BCa* = Sesgo corregido y acelerado con base en el bootstrapping basado en 10000 muestras.

Fuente: Datos de la encuesta.

Respecto a los padres, los estimadores predictivos de los factores de la violencia VFP se mostraron significativos. En cuanto a la predicción de la violencia física, el primer modelo se reportó significativo (*F* = 11.008, *p* = .001), explicando el 24.7% de la variación de la variable dependiente. La edad (*B* = .417, *p* = .009) y la puntuación AUDIT (*B* = .113, *p* = .011) son predictores significativos. En el caso del modelo 2 sobre la violencia verbal se mostró significativo (*F* = 11.561, *p* = .001), con una varianza explicativa de 25.7% sobre la variable dependiente, siendo la edad y la puntuación AUDIT predictores significativos. Esta misma situación se presentó en el modelo 3 de la violencia económica (*F* = 17.432, *p* = .001), aunque con una varianza explicativa de 34.2% sobre la variable dependiente. En cualquiera de las situaciones los resultados se interpretan a mayor edad e implicación con el uso de alcohol se ocasiona una elevada violencia de tipo física, verbal y económica contra los padres.

## DISCUSIÓN

Los hallazgos obtenidos en el presente estudio muestran que las prevalencias del consumo de alcohol en adolescentes de bachillerato presentan tendencia al aumento, además los porcentajes reportados del consumo por el sexo femenino en el presente estudio se encuentran por arriba de lo documentado por las encuestas del contexto mexicano y por estudios efectuados en similar población. El incremento del consumo en las mujeres pudiera atribuirse a los cambios en las normas sociales, en parte vinculados por los avances de igualdad de género, donde los comportamientos de riesgos ejercidos por el sexo femenino actualmente son aceptados y menos estigmatizados por la sociedad^(3–5,19)^. Además el incremento significativo del consumo de alcohol en ambos sexos pudiera explicarse por la pobre supervisión de venta y distribución de esta sustancia, aunado a la baja percepción de riesgo que tienen hacia el uso de alcohol^(3,19)^, algo que posee importantes implicaciones a nivel preventivo, por lo cual resalta la necesidad del trabajo en la adolescencia temprana (particularmente en adolescentes de bachillerato), ya que el inicio precoz del consumo de alcohol puede tener graves repercusiones en la salud y vida del adolescente.

En cuanto a la VFP por parte de los adolescentes hacia sus progenitores, se identificó que ambos sexos ejercen violencia, aunque en función del sexo del adolescente se esperaba que las mujeres se dirigieran con más violencia de tipo verbal y económica a las madres y los hombres ejercieran más violencia física a ambos progenitores^(21)^. En el presente estudio se encontró una importante tendencia a la violencia de tipo verbal, seguida de la económica y física por el sexo masculino, siendo los padres los principales receptores de los actos violentos. Los hallazgos apuntan que las familias mexicanas están sufriendo modificaciones en los estilos de crianza, pasando de ser familias autoritarias (donde el padre era el jefe de hogar) a familias permisivas-liberales, menos estrictas y con poco afecto (negligentes), en el que no se establecen normas claras ni hay un límite consistente de la autoridad^(22)^. En las familias permisivas y negligentes se ha observado que los hijos se caracterizan por estar sobreprotegidos, con poca empatía, se les otorga lo que piden sin exigir nada, por lo que, cuando no se satisface la demanda, los hijos asumen conductas antisociales y muchas veces conductas violentas de tipo verbal, física e incluso robos a los progenitores para obtener lo solicitado^(22,23)^.

El objetivo principal de esta investigación permitió analizar la relación y efecto del uso de alcohol sobre la VFP. Los hallazgos reproducen resultados de estudios previos realizados en diferentes contextos nacionales, aunque es de resaltar que el uso de alcohol por parte de los adolescentes mexicanos se relacionó de manera positiva con la violencia verbal y económica dirigida a las madres, mientras que a los padres se mostró correlación positiva con los tres factores de la VFP. Asimismo, el uso de alcohol a través de los modelos de regresión resultó explicativo de los factores de la VFP ejercida a las madres como a los padres, demostrando que el uso de esta sustancia aumenta la violencia a los progenitores. Por otro lado, la edad resultó un componente explicativo complementario únicamente de la violencia ejercida a los padres, indicando que, a mayor edad, más agresión.

A partir de los datos obtenidos de la relación entre el uso de alcohol y la VFP, se puede decir que los adolescentes que consumen alcohol muestran más violencia a ambos progenitores. En esta línea diversas investigaciones han señalado la implicación del uso de sustancias con la VFP y dentro de sus resultados muestran relaciones positivas consistentes entre el uso de sustancias y los factores de la VFP en población adolescente, aunque son escasos los estudios vinculados únicamente con el uso específico de alcohol^(24)^. En este sentido, la violencia ejercida por los adolescentes bajo los influjos de una sustancia puede ser explicada desde una razón instrumental o reactiva^(25)^. En la razón instrumental, cuando el adolescente tiene adicción a la sustancia, presenta búsqueda compulsiva de la misma, a pesar de sus consecuencias perjudiciales^(26)^. Al ser los progenitores la fuente económica para la compra del alcohol y ante la negativa del padre y la madre por la demanda del hijo e hija, se pueden desencadenar agresiones que posiblemente irán en aumento conforme aumenta la dependencia y la combinación de otras drogas, por lo que, el abuso de alcohol es un factor de riesgo para el comportamiento agresivo a el padre y a la madre^(25,26)^.

Mientras que en la razón reactiva puede explicarse como producto de la desinhibición verbal y conductual que ocasiona la sustancia en el adolescente, aumentando el riesgo de escalar a una agresión física^(25,26)^, de tal manera que los hallazgos sugieren que el consumo de alcohol es un desencadenante para la violencia que se vive en muchas familias, donde existe el miedo al hijo e hija y a las posibles lesiones físicas que puedan ocasionar, siendo en ocasiones silenciado debido a que se vuelven patrones que se toleran^(26)^.

Respecto a la particularidad de los tipos de violencia, los modelos de regresión mostraron que el uso de alcohol predice la violencia verbal y económica a las madres. Esto demuestra que las madres son vulneradas posiblemente al ser consideradas el sexo débil y por la cercanía que tienen con sus hijos, lo que las pone en una posición de riesgo^(27)^. A lo anterior también se le puede atribuir que en México existen cambios en los modelos familiares (disminución de familias nucleares), alrededor de 10 millones de familias son encabezadas por mujeres^(28)^, de tal manera que al ser las mujeres las jefas del hogar, requieren trabajar para sostener la familia y como consecuencia limitan el contacto con el hijo o la hija perdiendo el mantenimiento de la supervisión y autoridad, lo cual puede generar que el hijo adopte conductas no saludables como la ingesta de bebidas alcohólicas y al ser las madres la fuente de obtención de dinero para comprar alcohol y no acceder, pueden suscitar agresiones verbales e incluso robos a las madres^(27,28)^.

Asimismo, el uso de alcohol y la edad fueron predictores significativos de la violencia física, verbal y económica contra los padres. En relación con la violencia, los resultados concuerdan parcialmente con estudios previos del contexto mexicano donde se encontró predicción de la violencia verbal a los padres^(19)^, mientras que en Alemania se encontró predicción significativa por parte de los adolescentes varones sobre la violencia física ejercida a los progenitores^(29)^. Los resultados ponen de manifiesto que tanto el consumo de alcohol como la violencia tienen conexión significativa dentro del núcleo familiar y muchas veces son patrones que se repiten entre padres e hijos varones, siendo que actualmente el estilo de crianza permisivo y negligente por parte del padre puede estar afectando el comportamiento de los adolescentes, dado que cada vez los roles son más simétricos, perdiendo autoridad la figura paterna^(19,29)^.

En relación a la edad, los hallazgos aportan a los estudios previos, quienes señalan que el punto máximo de la violencia es entre los 14 y 16 años (adolescencia media), disminuyendo gradualmente conforme avanza la edad, no obstante, el presente estudio abordó adolescentes entre 14 y 19 años de edad, encontrando relación positiva entre la edad y la VFP, indicando que a mayor edad más violencia, hallazgos que muestran que la VFP no solo aparece durante la adolescencia sino también puede estar presente en otras etapas de la vida como la juventud^(19,29,30)^.

El estudio tiene algunas limitaciones. En primer lugar, se encuentra la selección de la muestra a través de un muestreo no probabilístico y en segundo lugar el tamaño muestral. Ambas limitaciones impiden la generalización de los resultados obtenidos y pudieran atribuir la baja varianza explicada sobre la variable dependiente. Otras limitantes observadas son la distribución no normal de las variables lo cual pudiera limitar la estimación predictiva del modelo sobre la variable dependiente, además la naturaleza del diseño, al ser de tipo transversal no puede establecer causalidad. Cabe mencionar que no se identificó el origen de los adolescentes, lo cual limita la representación significativa de cada región que integra el sur de México. En futuras investigaciones, se sugiere ampliar el tamaño muestral y aplicar un muestreo probabilístico para establecer una representación significativa en la muestra. También se recomienda establecer comparaciones del consumo de alcohol entre adolescentes menores de edad y adolescentes mayores de edad (o por grupo de edad) con la VFP, así como integrar otras variables que puedan influir en la aparición de la VFP, y con base a esto, ejecutar la construcción de un modelo con una fuerte rigurosidad metodológica.

## CONCLUSIÓN

La VFP aún es un tema oculto y estigmatizado entre las familias mexicanas. Los resultados confirman la relación y efecto del consumo de alcohol sobre la VFP, demostrando que la implicación que tiene el uso de alcohol por los adolescentes puede modificar la dinámica familiar y generar situaciones de conflicto como conductas violentas dentro del núcleo familiar (del hijo e hija a los progenitores).

Ante esto, el profesional de enfermería debe intervenir a través del diseño e implementación de intervenciones de enfermería dirigidas a prevenir o disminuir el uso de alcohol y los diferentes tipos de VFP. Además, el profesional de enfermería desde su campo de acción en el primer nivel de atención puede intervenir mediante la identificación de factores riesgo para el inicio del consumo de alcohol, así como detectar patrones de comportamiento violento del hijo e hija a el padre o a la madre, y con base a ello, promover estilos de creación protectores, fomentar en los adolescentes y padres de familia una comunicación asertiva y saludable para prevenir conductas de riesgos entre los integrantes del núcleo familiar.
